# Physiology Brings Relevance to Biological Research: A Vision for *Function*

**DOI:** 10.1093/function/zqae023

**Published:** 2024-05-06

**Authors:** David M Pollock

**Affiliations:** Section of CardioRenal Physiology & Medicine, Division of Nephrology, Department of Medicine, University of Alabama at Birmingham, Birmingham, AL 35223, USA

Scientific investigation has undergone profound changes since I began my research career over 40 yr ago. The pace of innovation has been marked by the introduction of numerous sophisticated techniques. As a graduate student, I witnessed the nascent use of polyacrylamide gels for measuring protein content while techniques we now consider routine like gene expression did not exist. Today, the use of genetically modified animals, advanced imaging, and multi-omic approaches that once seemed mere fantasies are now commonplace.

Historically, research disciplines were more isolated—only anatomists studied structures, biochemists focused on proteins and other biomolecules, pharmacologists developed and studied the behavior of drugs, and physiologists measured functions of complex living systems. Now, research transcends these boundaries, focusing on the most suitable methods to answer specific scientific questions, or at least that is the ideal we strive for.

While the technological revolution has allowed unprecedented insights into many biological processes, we should not forget the fundamental reasons for conducting research and that physiology plays an essential role in contextualizing the discoveries gained from these new technologies.

Understanding the interactions between genes, proteins, cells, and organ systems necessitates methodologies that underscore functional relevance. This requires the incorporation of physiology into the forefront of biological research in a holistic approach, which over 3 decades ago was the keystone of the “Systems Biology” revolution. To address the under-representation of what we might now call “integrated systems studies” in leading journals like *Nature, Cell*, and *Scien*ce, The American Physiological Society launched a new journal whose name, *Function*, reflects biological exploration in the broadest sense.

*Function* embraces emerging areas of physiological research across diverse areas such as immunology, neuroscience, the microbiome, circadian biology, and bioenergetics, among others. We encourage submissions not only from scientists who self-identify as traditional physiologists but also from all researchers who provide fresh perspectives on physiological systems.

In keeping with the changing world of scientific publishing, *Function* is an open-access journal with several innovative features that go beyond simple reporting of data as originally noted in our inaugural issue. One initiative includes wide use of *Perspectives* articles from leading experts to provide a broader view of the impact of the original research. Another category of article is *Function Focus*, where a limited study viewed as a major finding is reported without the full story found in a typical article. We are also including what we call *Evidence Reviews*, where the focus is on citing original work to avoid the ambiguity of who performed the work and what was actually done rather than making the reader search through other reviews only to come up empty handed.

*Function*’s editorial policy is designed to be author friendly. Our editorial team is also committed to make every effort to provide a clear decision after the initial review to avoid the all-too-common problem of requiring extensive revisions just to have the paper eventually rejected, as happens in so many leading journals. Although resubmission does not guarantee acceptance, we promise precise guidance on necessary revisions without demanding extensive new experiments.

We appreciate efforts of our current Executive Editors Donald Bers, Allen Cowley Jr, Annette Dolphin, Patricia Molina, Colin Nichols, and Ora Weisz, who have made significant contributions to the early success of the journal. These individuals are some of the world’s leading experts in cardiovascular signaling, mitochondrial energetics and related mechanisms, integrated blood pressure control, systems biology, ion channels, neuropharmacology, endocytosis and membrane transport, electrical systems in excitable tissues, state-of-the-art imaging, as well as metabolic and immune systems.

In the past few months, we have expanded the expertise on our leadership team with the addition of 5 new Executive Editors—Daniel Beard, Sue Bodine, Iain Drummond, Noriaki Emoto, and Eve Marder. Their expertise covers the areas of systems biology, neuromuscular function, aging, tissue regeneration, stem cell biology, cardiopulmonary disease, neurophysiology, and neural networks, among others. We also recruited 20 new board members who will join previous board members to expand the scope of the journal to include a range of credentials that include emerging areas related to organoids, microbiome, reproduction, and circadian biology, just to name a few.

Our expanded team of experts are committed to maintaining high editorial standards and ensuring the journal’s success. As the conductor of this world-class orchestra of international scientists, I am optimistic about our mission to position *Function* as a premier platform for groundbreaking research in the expansive field of physiological sciences.

Finally, heartfelt thanks to our previous editorial team members whose leadership helped *Function* achieve significant impact in its initial years, including high citation rates and notable scientific advancements. On a personal note, the contributions to *Function* from my own lab have been among the most impactful of my career, underscoring the journal’s importance in advancing physiological research. I hope that you will have a similar experience with this landmark new journal.
